# Hyporesponsiveness of natural killer cells and impaired inflammatory responses in critically ill patients

**DOI:** 10.1186/s12865-017-0231-y

**Published:** 2017-12-08

**Authors:** Minkyung Kim, Minjoo Kim, Hana Jeong, Jey Sook Chae, Young Sam Kim, Jae Gil Lee, Younsoo Cho, Jong Ho Lee

**Affiliations:** 1 Research Center for Silver Science, Institute of Symbiotic Life-TECH, Yonsei University, 50 Yonsei-ro, Seodaemun-gu, Seoul, 03722 Republic of Korea; 2 National Leading Research Laboratory of Clinical Nutrigenetics/Nutrigenomics, Department of Food and Nutrition, College of Human Ecology, Yonsei University, 50 Yonsei-ro, Seodaemun-gu, Seoul, 03722 Republic of Korea; 3 Department of Food and Nutrition, Brain Korea 21 PLUS Project, College of Human Ecology, Yonsei University, 50 Yonsei-ro, Seodaemun-gu, Seoul, 03722 Republic of Korea; 4 Department of Internal Medicine, Yonsei University College of Medicine, 50 Yonsei-ro, Seodaemun-gu, Seoul, 03722 Republic of Korea; 5 Department of Surgery, Yonsei University College of Medicine, 50 Yonsei-ro, Seodaemun-gu, Seoul, 03722 Republic of Korea; 6 Department of Nutrition, Yonsei University Health System, 50 Yonsei-ro, Seodaemun-gu, Seoul, 03722 Republic of Korea

**Keywords:** Cytokine, ICU, Inflammatory response, NK cell, PBMC

## Abstract

**Background:**

To investigate natural killer (NK) cell activity, circulating cytokine level and peripheral blood mononuclear cell (PBMC) cytokine production status in critically ill patients.

**Methods:**

Blood samples were collected <24 h after admission from 24 intensive care unit (ICU) patients and 24 age-, sex-, and body mass index (BMI)-matched healthy controls. Serum cytokine concentrations and cytokine production by PBMCs and lipopolysaccharide (LPS)-stimulated PBMCs were measured.

**Results:**

The ICU group showed lower NK cell activity than the controls under all conditions and an absence of interferon (IFN)-γ. After adjusting for triglycerides, LDL- and HDL-cholesterol, and glucose, the ICU group exhibited lower serum levels of albumin and interleukin (IL)-12 and higher leukocyte counts and hs-CRP and IL-6 levels than the controls. Non-stimulated PBMCs from ICU patients secreted significantly greater amounts of IL-6 and IL-1β than the controls; however, the production of IL-6, TNF-α and IL-1β in response to LPS stimulation was significantly lower in the ICU group.

**Conclusions:**

Significant reductions in NK cell activity and serum IL-12 level, an absence of serum IFN-γ, and decreased cytokine production from LPS-stimulated PBMCs indicate the hyporesponsiveness of NK cells and an impaired early phase inflammatory response in critically ill patients (ClinicalTrials.gov NCT02565589:). Retrospectively registered; October 1, 2015.

## Background

The host response to systemic infection and acute inflammatory states is characterized by fast and marked alterations in innate and adaptive immunity [1]. Critically ill patients with various conditions, including sepsis, trauma, burns, hemorrhagic shock, and severe surgery, show exacerbated production of proinflammatory mediators, a status that is called systemic inflammatory response syndrome (SIRS) [2]. In response to this proinflammatory state, there is a compensatory release of anti-inflammatory substances, a status that is called compensatory anti-inflammatory response syndrome (CARS) [2]. In general, proinflammatory reactions are thought to be responsible for collateral tissue damage in severe sepsis, whereas anti-inflammatory responses are implicated in enhanced susceptibility to secondary infections [3]. Accordingly, CARS often leads to suppression of the immune system, which makes patients vulnerable to nosocomial infections [4] and leads to increased morbidity and mortality in the ICU [2].

Critical illnesses can deregulate every component of the immune response [5–7]. In patients infected following surgery, trauma, or burn, circulating monocytes exhibit reduced production of IL-12 [8, 9]. Decreased monocyte interleukin (IL)-12 production was significantly correlated with adverse clinical outcomes [10]. Several circulating cytokines and cytokine production by peripheral blood mononuclear cells (PBMCs) play vital roles in critically ill patients [11–13]. The stimulated level of whole blood cytokine production is theoretically a good indicator of host immunity because it reflects actual cell function, such as their ability to produce key cytokines involved in host defense [14]. In a prospective cohort clinical study, the number of CD14^+^ monocytes producing IL-12, tumor necrosis factor (TNF)-α, and IL-6 after lipopolysaccharide stimulation was 40% to 70% lower in trauma patients than in healthy control subjects [8]. Critically ill pediatric patients with persistently low stimulated TNF-α production are more likely to acquire life-threatening infections, and treatment caused rapid improvement in stimulated TNF-α production, which was associated with the prevention of nosocomial infections [15, 16].

NK cells are crucial components of the innate immune system, representing 10% of the cells in the total PBMC population of circulating human lymphocytes, which is the third largest lymphocyte population following B and T cells [17]. NK cells contribute to the immune-inflammatory reaction by producing many cytokines, particularly interferon (IFN)-γ, a potent immune-stimulating cytokine [18]. NK cells have a protective role during infection but can also be harmful during systemic inflammation and are associated with lethality and experimental sepsis [19, 20]. In a comparison of ICU patients with severe sepsis or septic shock or non-septic SIRS and healthy controls, septic patients exhibited reduced IFN-γ production by NK cells, whereas SIRS patients exhibited increased IFN-γ production compared to that of sepsis patients or healthy controls [21]. However, few studies have compared NK cell activity in non-septic, critically ill patients with that in healthy controls. Therefore, the aim of this study was to investigate NK cell activities, circulating cytokine levels, non-stimulated PBMC cytokine production, lipopolysaccharide (LPS)-stimulated PBMC cytokine production, and nutritional status in the early phase (< 24 h after ICU admission) in critically ill patients in comparison with age-, sex-, and body mass index (BMI)-matched healthy controls.

## Methods

### Study design

Critically ill patients (ICU group, *n* = 24) were enrolled between April 2015 and July 2015 from Yonsei University Severance Hospital after admission to the ICU. For validation of the experimental findings, age-, sex- and BMI-matched healthy subjects were enrolled as controls from a local health evaluation center during the same period (ClinicalTrials.gov: NCT02565589; http://www.clinicaltrials.gov). Among the critically ill patients, 16 patients had pulmonary disease, and 8 patients had trauma. Disease severity was evaluated with the Acute Physiology and Chronic Health Evaluation (APACHE) II score [22]. All of the patients were treated according to appropriate guidelines [23, 24]. Written informed consent was provided from the healthy participants and from a close family member for the ICU group. Institutional Review Board at Yonsei University Severance Hospital approved the study protocol, which was conducted according to the Declaration of Helsinki.

### Anthropometric parameters, biochemical assessments, and cytokine and NK cell activity assays

Anthropometric parameters, serum lipid profiles, glucose, nutritional status, liver and renal function tests, cytokine levels in serum and PBMC supernatants, and NK cell activity were analyzed as previously described [25]. Detailed information about assessments of cytokine levels in serum and PBMC supernatants, and NK cell activity were described in supplementary information (See Additional File 1).

### Statistical analysis

SPSS version 21.0 (IBM/SPSS Corp. Chicago, IL) was used for statistical analysis. Comparisons between the control and ICU groups were performed using the Chi-square test for categorical variables and independent *t*-tests for continuous variables. Adjustment for confounding variables was performed using an ANCOVA analysis. *p*-values under 0.05 were regarded as statistically significant, and *p*-values for skewed variables were analyzed using log-transformed data. The results are presented as the means ± standard errors, and untransformed values are presented for descriptive purposes. The relationships between variables were examined using Pearson’s correlation coefficient and visualized as a heat map.

## Results

### Clinical characteristics and serum cytokine levels

Compared with the age-, sex-, and BMI-matched healthy controls, the ICU group exhibited a significant decrease in triglycerides, total-, LDL- and HDL-cholesterol, albumin, prealbumin, IL-12, and IFN-γ levels and a significant increase in serum glucose, leukocyte count, and the serum levels of hs-CRP, IL-6 and IL-1β (Table 1). The ICU group exhibited an absence of IFN-γ (below the detection limit), in contrast to the control group. The ICU group displayed lower serum levels of albumin (*p* < 0.001), prealbumin (*p* = 0.010), and IL-12 (*p* = 0.012) and a higher leukocyte count (*p* = 0.012), hs-CRP (*p* < 0.001), and IL-6 (*p* < 0.001) levels than the control group after adjustment for triglycerides, LDL- and HDL-cholesterol and glucose levels.

**Table 1:** Clinical characteristics in control and ICU patient groups

	Control group (*n* = 24)	ICU patient (*n* = 24)	*p*_0_	*p*_1_
Age (year)	64.5 ± 2.05	64.6 ± 3.06	0.991	
Male/Female *n*, (%)	18 (75.0) / 6 (25.0)	18 (75.0) / 6 (25.0)	1.000	
BMI (kg/m^2^)	22.3 ± 0.40	22.4 ± 0.77	0.977	
Height (cm)	163.7 ± 1.82	166.7 ± 1.41	0.201	
Weight (kg)	64.0 ± 2.06	62.5 ± 2.69	0.669	
Glucose (mg/dL)^∮^	97.0 ± 4.95	211.4 ± 38.1	<0.001	
Triglyceride (mg/dL)^∮^	135.5 ± 9.41	104.8 ± 8.85	0.018	
Total-cholesterol (mg/dL)^∮^	216.3 ± 10.9	111.6 ± 7.16	<0.001	
LDL-cholesterol (mg/dL)^∮^	129.2 ± 10.1	61.4 ± 5.81	<0.001	
HDL-cholesterol (mg/dL)^∮^	59.8 ± 3.63	29.2 ± 2.85	<0.001	
GOT (IU/L)^∮^	28.5 ± 2.34	35.8 ± 6.02	0.708	0.155
GPT (IU/L)^∮^	24.1 ± 1.91	22.2 ± 4.03	0.097	0.581
γ-GTP (U/L)^∮^	34.8 ± 8.53	51.4 ± 15.9	0.967	0.520
BUN (mg/dL)^∮^	15.1 ± 0.58	19.1 ± 2.54	0.811	0.294
Creatinine (mg/dL)^∮^	0.82 ± 0.03	0.83 ± 0.07	0.556	0.567
Albumin (mg/dL)^∮^	4.94 ± 0.11	2.77 ± 0.11	<0.001	<0.001
Prealbumin (mg/dL)^∮^	25.6 ± 0.97	10.7 ± 0.97	<0.001	0.010
Leukocyte count (×10^3^/μL)^∮^	5.65 ± 0.25	10.8 ± 0.92	<0.001	0.012
hs-CRP (mg/L)^∮^	1.01 ± 0.29	113.1 ± 15.1	<0.001	<0.001
IL-12 (pg/mL)	41.5 ± 7.78	2.49 ± 1.59	<0.001	0.012
IFN-γ (pg/mL)	14.8 ± 2.61	0.00 ± 0.00	<0.001	0.031
TNF-α (pg/mL)^∮^	11.7 ± 2.27	6.49 ± 0.70	0.094	0.816
IL-6 (pg/mL)^∮^	2.55 ± 0.28	56.4 ± 16.9	<0.001	<0.001
IL-1β (pg/mL^∮*∮*^	0.56 ± 0.05	2.54 ± 0.15	0.001	0.091

Mean ± SE.^∮^tested by logarithmic transformation. *p*_0_: derived from independent *t*-test. *p*_1_: adjusted for glucose, triglyceride, LDL-cholesterol, and HDL-cholesterol

### NK cell activity and PBMC cytokine production in response to LPS in ICU patients

NK cell activities determined in all conditions (E:*T* = 10:1, 5:1, 2.5:1 and 1.25:1) were significantly lower in the ICU group than the control group (Table 2). Cytokine levels measured in cultured PBMC supernatants following LPS stimulation (0, 5, 10 ng/mL) for the control and ICU groups are shown in Table 2. Non-stimulated PBMCs from ICU patients secreted significantly greater amounts of IL-6 and IL-1β into the culture media than those from healthy controls. Both the control and patient groups showed increases in the levels of TNF-α, IL-6 and IL-1β after LPS stimulation. However, the production of IL-6 and TNF-α in response to LPS stimulation (5 or 10 ng/mL) was lower in the ICU group than the control group. Similarly, the production of IL-1β in response to LPS stimulation (10 ng/mL) was lower in the ICU group compared to the control group (*p* = 0.001).

**Table 2:** Natural killer cell activities and cytokine production from PBMCs

	Control group (*n* = 24)	ICU patient (*n* = 24)	*p*
NK cell activity			
NK cell activity E:T = 10:1 (%)^∮^	27.4 ± 2.37	16.4 ± 1.99	0.002
NK cell activity E:*T* = 5:1 (%)^∮^	20.8 ± 1.85	13.1 ± 2.15	0.006
NK cell activity E:*T* = 2.5:1 (%)^∮^	20.1 ± 2.20	13.0 ± 1.94	0.011
NK cell activity E:*T* = 1.25:1 (%)^∮^	23.1 ± 2.24	15.1 ± 2.19	0.013
Non-stimulated PBMC			
TNF-α (pg/mL)^∮^	2.03 ± 0.31	5.26 ± 1.48	0.103
IL-6 (pg/mL)^∮^	12.1 ± 3.57	19.9 ± 3.29	0.039
IL-1β (pg/mL)^∮^	0.70 ± 0.24	2.58 ± 0.51	0.002
PBMC (LPS 5 ng/mL)			
TNF-α (pg/mL)^∮^	299.7 ± 42.5	173.0 ± 39.2	0.007
IL-6 (pg/mL)^∮^	2045.3 ± 341.9	685.9 ± 238.8	0.025
IL-1β (pg/mL)^∮^	22.5 ± 5.90	18.0 ± 6.79	0.304
PBMC (LPS 10 ng/mL)			
TNF-α (pg/mL)^∮^	322.3 ± 49.2	210.5 ± 57.7	0.015
IL-6 (pg/mL)^∮^	2479.2 ± 369.2	498.9 ± 108.4	<0.001
IL-1β (pg/mL)^∮^	24.7 ± 3.77	15.2 ± 4.92	0.001

Mean ± SE.^∮^tested by logarithmic transformation. *p*: derived from independent *t*-test

### Correlations among age, BMI, leukocyte count, serum levels of hs-CRP, albumin, and prealbumin, and PBMC cytokine production

**Fig. 1:**
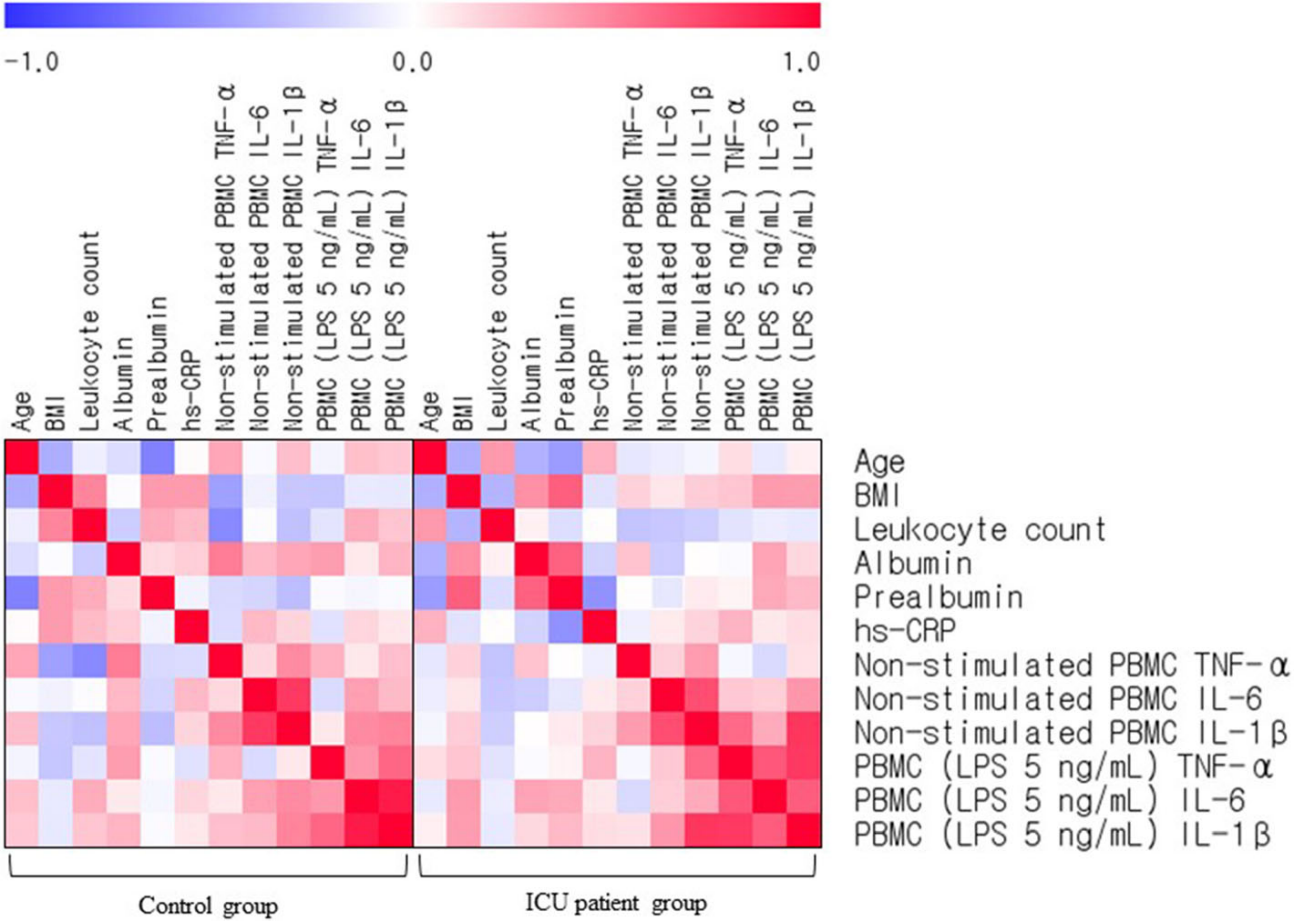
Correlation matrix among clinical and inflammatory parameters in control and ICU patient groups. Correlations were obtained by deriving Pearson’s correlation coefficient. *Red* denotes a positive correlation, and *blue* denotes a negative correlation

In the ICU group, there were significant positive correlations among BMI, serum albumin, and serum prealbumin. Serum prealbumin was weakly and negatively correlated with age (*r* = −0.480, *p =* 0.021), moderately correlated with hs-CRP (*r* = −0.530, *p =* 0.009), and positively correlated with PBMC TNF-α production after LPS stimulation (5 ng/mL) (*r* = 0.625, *p =* 0.002), which was also associated with BMI (*r* = 0.567, *p =* 0.007) and serum albumin (*r* = 0.633, *p =* 0.002). Non-stimulated PBMC IL-6 was positively associated with non-stimulated PBMC IL-1β (*r* = 0.695, *p <* 0.001), which had a strong positive correlation with LPS (5 ng/mL)-stimulated PBMC IL-1β (*r* = 0.789, *p <* 0.001) and a low positive correlation with TNF-α (*r* = 0.468, *p =* 0.033). There were positive correlations among LPS (5 ng/mL)-stimulated PBMC IL-1β, LPS-stimulated PBMC TNF-α, and LPS-stimulated IL-6 (Fig. 1).

In the control group, serum prealbumin was negatively correlated with age (*r* = −0.611, *p =* 0.002). BMI was positively and weakly correlated with the leukocyte count (*r* = 0.482, *p =* 0.017) and negatively correlated with non-stimulated PBMC TNF-α (*r* = −0.463, *p =* 0.026), which was negatively associated with leukocyte count (*r* = −0.571, *p =* 0.004) and positively associated with serum albumin (*r* = 0.510, *p =* 0.013). Non-stimulated PBMC TNF-α exhibited positive but weak correlation with non-stimulated PBMC IL-1β (*r* = 0.458, *p =* 0.028). Non-stimulated PBMC IL-6 was strongly and positively correlated with non-stimulated PBMC IL-1β (*r* = 0.779, *p <* 0.001), which showed positive but weak correlation with LPS (5 ng/mL)-stimulated PBMC IL-1β (*r* = 0.484, *p =* 0.017) and IL-6 (*r* = 0.455, *p =* 0.025). There were strong positive correlations among LPS (5 ng/mL)-stimulated PBMC IL-1β, TNF-α, and IL-6 (Fig. 1).

## Discussion

This study showed that NK cell activities were significantly decreased under all conditions (E:*T* = 10:1, 5:1, 2.5:1, and 1.25:1) in the early phase in critically ill patients compared with those in age-, sex-, and BMI-matched healthy controls. This finding is in accordance with another recent study suggesting that NK cells and monocytes exhibit hyporesponsiveness during critical illness [26]. NK cells have the capacity to induce apoptosis or cell lysis in tumor cells, virus-infected cells, and intracellular parasites [27]. NK cells play an immuno-modulatory role by secreting several cytokines, including IFN-γ, which activates many key pathways related to antiviral functions [17]. IL-12 is involved in stimulation of IFN-γ production from T cells and NK cells [28]. Thus, the large reduction in NK cell activity and serum IL-12 levels as well as the absence of serum IFN-γ observed in the ICU group in this study indicate NK cell hyporesponsiveness during the early phase in critically ill patients.

Increased IL-12 production may be associated with increased cellular immunity in critically ill patients. For instance, LPS-stimulated PBMCs from survivors with severe sepsis produced more IL-12 and less IL-10 than those from nonsurvivors [12]. Similarly, the LPS-stimulated PBMCs from survivors with septic shock produced more TNF-α than those from nonsurvivors [29]. In this study, serum TNF-α and TNF-α production by non-stimulated PBMCs were not significantly different between the control and ICU groups, while TNF-α production in response to LPS stimulation (5, 10 ng/mL) was significantly lower in the ICU group than the healthy controls. Additionally, TNF-α production in response to LPS stimulation (5 ng/mL) in the ICU group was positively correlated with nutritional status, including BMI and the serum concentrations of albumin and prealbumin. However, this association was not observed in the healthy controls. Patients showing impaired TNF-α production in leukocytes upon ex vivo LPS stimulation have been reported to exhibit an increased risk of developing nosocomial infections [30]. Therefore, the positive correlation between TNF-α production in response to LPS stimulation and nutritional status and the negative correlation between hs-CRP and prealbumin observed in the ICU group in this study suggest the importance of nutritional therapy in critically ill patients to improve health outcomes, including cellular immunity [31–34].

Serum hs-CRP levels have been reported to positively correlate with serum IL-6, which is always increased in acutely ill patients with severe sepsis [26, 35]. Similarly, the ICU group in this study showed higher serum levels of hs-CRP and IL-6 and leukocyte count than the control group. Furthermore, the production of IL-6 and IL-1β by non-stimulated PBMCs was higher in the ICU group, although the production of IL-6 and IL-1β in response to LPS stimulation was lower in the ICU group. There is an age-related decline in cytokine production by monocytes, particularly for the proinflammatory mediators IL-6, TNF-α, and IL-1β [36–40]. Indeed, older patients are known to exhibit less effective neutrophil activity [37] and decreased NK cell cytotoxicity and macrophage function [39, 41]. However, the lower cytokine production following LPS stimulation in the ICU group could have resulted from critical illness, including pneumonia and trauma, rather than age, sex, or low body weight, as the healthy controls were age-, sex-, and BMI-matched to the critically ill patients in this study. In this study, there was a closer correlation between IL-6, TNF-α, and IL-1β levels from LPS-stimulated PBMCs from the healthy controls compared with the ICU group.

The limitations of this study must be addressed. First, the sample size of 24 critically ill patients warrants confirmation in larger trials. Second, the study was an observational study, in which elucidating the cause and effect relationships of specific (immunological and inflammatory) mechanisms was not possible. Finally, our results do not directly demonstrate that NK cell hyporesponsiveness in the ICU group was significantly correlated with decreased IL-12 and IFN-γ. Despite these limitations, we observed greater reductions in NK cell activities and serum IL-12 levels and an absence of serum IFN-γ in the early phase in critically ill patients than in age-, sex-, and BMI-matched healthy controls. We also detected a decrease in serum albumin and prealbumin and an increase in the leukocyte count, serum IL-6, and hs-CRP. Furthermore, in contrast to the increased production of IL-6 and IL-1β from non-stimulated PBMCs, the decreased production of IL-6, TNF-α, and IL-1β from LPS-stimulated PBMCs in the ICU group suggest an impaired inflammatory response in critically ill patients.

## Conclusions

This study investigated NK cell activities, circulating cytokine levels and PBMC cytokine production status in critically ill patients. The ICU group showed lower NK cell activities and serum levels of albumin and IL-12 and higher leukocyte counts and hs-CRP and IL-6 levels than the controls. Non-stimulated PBMCs from ICU patients secreted significantly higher amounts of IL-6 and IL-1β than those from controls, while production of IL-6 and TNF-α in response to LPS stimulation (5 or 10 ng/mL) was lower in the ICU group. Similarly, the production of IL-1β in response to LPS stimulation (10 ng/mL) was also significantly lower in the ICU group. Our findings suggest that great reductions in NK cell activities and serum IL-12 levels, an absence of serum IFN-γ, and decreased cytokine production from LPS-stimulated PBMCs could indicate the hyporesponsiveness of NK cells and an impaired inflammatory response in the early phase of critical illness.

## Abbreviations

BMI: Body mass index
BUN: Blood urea nitrogen
CARS: Compensatory anti-inflammatory response syndrome
GOT: Glutamic oxaloacetic transaminase
GPT: Glutamic pyruvate transaminase
hs-CRP: High-sensitivity C-reactive protein
ICU: Intensive care unit
IFN: Interferon
IL: Interleukin
LPS: Lipopolysaccharide
NK: Natural killer
PBMC: Peripheral blood mononuclear cell
SIRS: Systemic inflammatory response syndrome
TNF: Tumor necrosis factor
γ-GTP: Gamma-glutamyl transpeptidase
